# Congenital myasthenic syndrome with mild intellectual disability caused by a recurrent *SLC25A1* variant

**DOI:** 10.1038/s41431-019-0506-2

**Published:** 2019-09-16

**Authors:** Sunitha Balaraju, Ana Töpf, Grace McMacken, Veeramani Preethish Kumar, Astrid Pechmann, Helen Roper, Seena Vengalil, Kiran Polavarapu, Saraswati Nashi, Niranjan Prakash Mahajan, Ines A. Barbosa, Charu Deshpande, Robert W. Taylor, Judith Cossins, David Beeson, Steven Laurie, Janbernd Kirschner, Rita Horvath, Robert McFarland, Atchayaram Nalini, Hanns Lochmüller

**Affiliations:** 1 Department of Clinical Neurosciences, University of Cambridge School of Clinical Medicine, Cambridge, UK; 2 Institute of Genetic Medicine, Newcastle University, Newcastle upon Tyne, UK; 3 Department of Neurology, National Institute of Mental Health and Neurosciences, Bengaluru, India; 4 Department of Clinical Neurosciences, National Institute of Mental Health and Neurosciences, Bengaluru, India; 5 Department of Neuropediatrics and Muscle Disorders, Medical Center–University of Freiburg, Faculty of Medicine, Freiburg, Germany; 6 Department of Paediatrics, Heartlands Hospital, Birmingham, UK; 7 Department of Medical and Molecular Genetics, School of Basic and Medical Biosciences King’s College London, London, UK; 8 Clinical Genetics Unit, Guys and St Thomas’ NHS Foundation Trust, London, UK; 9 Wellcome Centre for Mitochondrial Research, Institute of Neuroscience, Newcastle University, Newcastle upon Tyne, UK; 10 Neurosciences Group, Nuffield Department of Clinical Neuroscience, Weatherall Institute of Molecular Medicine, University of Oxford, Oxford, UK; 11 Centro Nacional de Análisis Genómico (CNAG-CRG), Center for Genomic Regulation, Barcelona Institute of Science and Technology (BIST), Barcelona, Catalonia Spain; 12 Children’s Hospital of Eastern Ontario Research Institute; Division of Neurology, Department of Medicine, The Ottawa Hospital; Brain and Mind Research Institute, University of Ottawa, Ottawa, ON Canada

**Keywords:** Next-generation sequencing, Genetic testing

## Abstract

Congenital myasthenic syndromes (CMS) are a clinically and genetically heterogeneous group of disorders caused by mutations which lead to impaired neuromuscular transmission. *SLC25A1* encodes a mitochondrial citrate carrier, associated mainly with the severe neurometabolic disease combined D-2- and L-2-hydroxyglutaric aciduria (D/L-2-HGA). We previously reported a single family with a homozygous missense variant in *SLC25A1* with a phenotype restricted to relatively mild CMS with intellectual disability, but to date no additional cases of this CMS subtype had been reported. Here, we performed whole exome sequencing (WES) in three additional and unrelated families presenting with CMS and mild intellectual disability to identify the underlying causative gene. The WES analysis revealed the presence of a homozygous c.740G>A; p.(Arg247Gln) missense *SLC25A1* variant, the same *SLC25A1* variant as identified in the original family with this phenotype. Electron microscopy of muscle from two cases revealed enlarged and accumulated mitochondria. Haplotype analysis performed in two unrelated families suggested that this variant is a result of recurrent mutation and not a founder effect. This suggests that p.(Arg247Gln) is associated with a relatively mild CMS phenotype with subtle mitochondrial abnormalities, while other variants in this gene cause more severe neurometabolic disease. In conclusion, the p.(Arg247Gln) *SLC25A1* variant should be considered in patients presenting with a presynaptic CMS phenotype, particularly with accompanying intellectual disability.

## Introduction

Congenital myasthenic syndromes (CMS) are a heterogeneous group of disorders caused by mutations leading to impairment of neuromuscular transmission. Most often, these disorders arise from mutations affecting postsynaptic components of the neuromuscular junction (NMJ). CMS due to mutations affecting the motor nerve terminal comprise a rarer subset; they are however, increasingly recognised, and the majority of the recently discovered CMS genes result in presynaptic NMJ defects [1].

*SLC25A1* is a mitochondrial citrate carrier which mediates the exchange of citrate/isocitrate with cytosolic malate [2]. Variants in the *SLC25A1* gene are associated with severe neurometabolic disease [3–5]. We previously identified a homozygous c.740G>A; p.(Arg247Gln) missense variant in *SLC25A1* in a British sib-pair presenting with a mild form of CMS with intellectual disability [6]. Here we report three additional unrelated CMS families carrying the same missense variant in *SLC25A1*, presenting with a similar phenotype, confirming the genotype–phenotype association.

## Materials and methods

### Patients

Patients were identified and fully investigated with standard clinical, electrophysiological and myopathological examinations at specialised neurology and neuromuscular clinics, namely Department of Neurology, National Institute of Mental Health and Neuroscience, India, Department of Neuropediatrics and Muscle Disorders, Freiburg University, Germany and the NHS Highly Specialised Mitochondrial disease service, Newcastle Upon Tyne hospitals NHS Foundation Trust, UK. Written consent was obtained for all participants to publish their clinical photographs. DNA samples were submitted to the Newcastle MRC Centre Biobank for Neuromuscular Diseases for which ethical approval was granted by the NRES Committee North East—Newcastle & North Tyneside 1 (reference 08/H0906/28). We have conducted the research in accordance with the Declaration of Helsinki.

### Whole exome analysis (WES) and Haplotype analysis

WES was performed by the Genomics Platform at the Broad Institute of MIT and Harvard, Cambridge, USA. Libraries were created with an Illumina exome capture (38 Mb target) and sequenced with a mean target coverage of >80×. Exome data was then processed at the Centro Nacional de Análisis Genómico (CNAG), Barcelona, Spain, and variant prioritization was carried out on the Genome–Phenome Analysis Platform. Standard filtering criteria were applied, including minor allele frequency of 1% and high to moderate effect on protein structure (i.e. nonsense, splice site, frame shift, in-frame and non-synonymous variants), and using a gene list of 416 genes known to be associated with neuromuscular disease. The identified *SLC25A1* variant has been submitted to ClinVar ((https://www.ncbi.nlm.nih.gov/clinvar/) under accession number: SCV000853306.1). Genomic data for patients 1/1, 2/1 and 2/2 were analysed for runs of homozygosity (RoH) using PLINK (http://zzz.bwh.harvard.edu/plink/ibdibs.shtml) and the respective haplotypes were identified manually.

## Results

### Clinical description

**Fig. 1:**
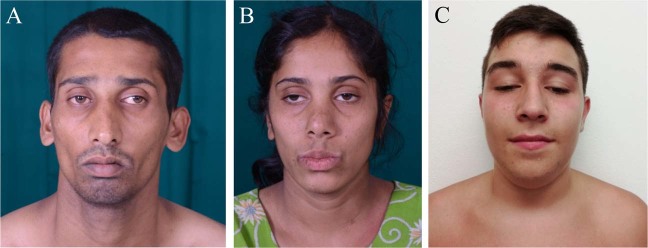
Representative clinical pictures of patients with a pathogenic *SLC25A1* variant. **a** Symmetrical facial weakness and ophthalmoparesis (patient 2/1). **b** Ptosis and mild facial weakness (patient 2/2). **c** Severe ptosis with compensatory over-activation of frontalis and mild facial weakness (patient 3/1)

We identified six patients from four families of different ethnicity (British, Indian, Greek and Pakistani) (Table 1 and Fig. 1). Three families were consanguineous. All patients developed a non-progressive proximal weakness in early infancy, which fluctuated with physical activity and was clearly fatigable on examination. Some degree of involvement of extraocular muscles was found in all patients, which ranged from severe bilateral ptosis (Fig. 1c, patient 3/1) to subjective diplopia only (patient 1/2). Respiratory function was normal in all cases. In addition, all patients had mild intellectual disability. No symptoms of autonomic dysfunction were reported by any of the patients. MRI brain was performed in four cases and was normal in all. Serum lactate was normal at rest in all, but in two cases showed increase after exercise. Two patients had functional benefit from acetylcholinesterase (AChE) inhibitor treatment, but in other cases it was not beneficial despite adequate dosing. One case was treated with 3, 4-diaminopyridine and responded well to the treatment, in-keeping with a presynaptic NMJ defect (Table 1).

**Table:** Summary of the clinical, neurophysiological and biochemical features of the cases

	Previous study		Current study			
Family/patient/age at exam/sex	1/1/37/M	1/2/23/F	2/1/26/M	2/2/25/F	3/1/14/M	4/1/14/F
Ethnic origin	British	British	Indian	Indian	Greek Pomak	Pakistani
Consanguinity	Yes	Yes	Yes	Yes	No	Yes
Age of onset	Infancy	Infancy	Infancy	Infancy	Infancy	Infancy
CMS-associated features						
Ocular involvement	Mild bilateral ptosis, no ophthal	Diplopia, no ptosis	Mild ophthal and ptosis	Mild ophthal and ptosis	Marked ptosis, mild ophthal	Mild ptosis, diplopia
Bulbar involvement	Yes	No	No	No	Yes	Yes
Weakness distribution	Proximal, UL = LL	Proximal, UL = LL, NF weakness	Proximal, UL = LL	Proximal, UL = LL	Proximal, UL = LL	Proximal, LL > UL, NF weakness
Fatigability	Yes	Yes	Yes	Yes	Yes	Yes
Treatment; response	AChEI—no response; 3,4-DAP—good response	AChEI—some response	AChEI and salbutamol—no response	AChEI and salbutamol—no response	AChEI—some response	AChEI—no response
Additional clinical features						
Intellectual disability	Mild, attended mainstream school	Yes, attended school with additional support needs	Unable to read/write/calculate Binet-Kamat IQ score: 45	Unable to read/write	Yes, attended school with additional support needs	Mild, attended mainstream school
Dysmorphism	No	No	Elongated facies, prominent ears, high arched palate	Elongated facies, prominent ears	No	No
Other	Pes cavus	Bicuspid AV and MV prolapse	Hypermobility, increase in lactate with exercise	Hyperextensible joints, chin fasciculations	Pes planovalgus	Weakness exacerbated by heat and cold. Marked increase in lactate with exercise
Neurophysiology						
Repetitive nerve stimulation	No dec on 3 Hz stimulation, no inc after 20 s MVC	Not done	Decrement on 3 Hz stimulation (MVC not performed)	Decrement on 3 Hz stimulation (MVC not performed)	No dec on 3 Hz stimulation, no inc after 20 s MVC	No dec on 3 Hz stimulation (MVC not performed)
Single fibre EMG	Increased jitter and block	Not done	Not done	Not done	Not done	Jitter within normal limits
Additional analysis						
Muscle biopsy	Normal COX staining, enlarged mitochondria and increased in number on EM	Not done	Not done	Not done	Fibre size variation, few central cores; sporadically accumulated mitochondria in EM	Mildly atrophic fibres, normal COX staining, mild reduction in complex 1 activity
Urine 2-hydroxyglutarate	Normal	Normal	Not done	Not done	Normal	Not done
MRI brain	Normal	Normal	Not performed	Normal	Not performed	Normal

3,4-*DAP* 3,4-diaminopyridine, *AChEI* acetylcholinesterase inhibitor, *COX* cytochrome c oxidase, *Dec* decrement of compound muscle action potential, *EM* electron microscopy, *EMG* electromyography, *Inc* increment of compound muscle action potential, *LL* lower limb, *MVC* maximum voluntary contraction, *NF* neck flexor weakness, *Ophthal* ophthalmoplegia, *RNS* repetitive nerve stimulation, *UL* lower limb

Repetitive nerve stimulation (RNS) showed decrement in two patients at 3 Hz stimulation, and significantly increased jitter on single fibre electromyography in five patients.

Muscle biopsy was performed in three cases and showed non-specific myopathic features in all. In addition, on electron microscopy two cases showed enlarged and accumulated mitochondria.

### Genetic analysis

After WES variant filtering, the same homozygous missense variant hg19 chr22:g.19164098C>T; c.740G>A; p.(Arg247Gln) in *SLC25A1* (GenBank NM_005984.5) was identified in all patients. Sequencing data for patients 1/1 (British origin), 2/1 and 2/2 (Indian origin) were analysed to identify possible RoH around the *SLC25A1* variant. As expected due to their consanguinity, patients 2/1 and 2/2 shared a large RoH of 1.43 Mb (from chr22:18870865 to 20325552), whereas patient 1/1 had a much smaller RoH of 0.15 Mb (from chr22:19148244 to 19299703). The genotypes within the shared region between the two families differ in at least five homozygous calls (i.e. hg38 chr22: g.19177322A>C; rs3213491, hg38 chr22:g.19199939G>A; rs2236760, hg38 chr22:g.19199940C>G; rs2236761, hg38 chr22:g.19208992G>A; rs1060376 and hg38 chr22:g.19258298A>G; rs3788295), suggesting that they represent two distinct haplotypes.

## Discussion

Pathogenic variants in *SLC25A1* cause combined D-2- and L-2-hydroxyglutaric aciduria (D/L-2-HGA), a severe metabolic neurodevelopmental disorder with early lethality [3–5]. We previously reported that the homozygous p.(Arg247Gln) missense variant caused a CMS phenotype but without systemic manifestations of a mitochondrial disease [6]. Here, we report three additional CMS families of different ethnicity harbouring the same homozygous missense variant. Interestingly, the haplotype of the Indian and British families [6] were distinct, suggesting this variant is not seen as a result of a founder effect but rather to recurrent mutation.

Clinically these families had early onset, fatigable ocular, bulbar and proximal muscle weakness, which are clinical hallmarks of impaired neuromuscular transmission. In addition, cognitive impairment was found in all cases. Several presynaptic CMS genes including *SNAP25B* [7], *MUNC13* [8], *MYO9A* [9], *SLC18A3* [10] and *SLC5A7* [11], as well as mutations in *DPAGT1* [12], are known to be associated with cognitive and behavioural problems, which may reflect the importance of these genes in central as well as peripheral synapses. Our findings suggest that *SLC25A1* and in particular the p.(Arg247Gln) variant should also be considered in CMS cases with intellectual disability.

Ptosis, ophthalmoplegia and fatigable muscle weakness can also occur in mitochondrial disease along with many other severe systemic symptoms [13]. Unlike the typical clinical progression in mitochondrial disorders, the muscle weakness in our cohort was non-progressive. Pathogenic variants in *SLC25A1* which cause D/L-2-HGA are typically in amino acid residues which are crucial for protein activity, whereas the variant documented here was shown to result in reduced carrier activity with compensatory increase in protein expression [6, 14]. Consistent with this, all patients were found to have normal urinary 2-HG levels. In addition, muscle biopsy showed subtle mitochondrial abnormalities on electron microscopy, but normal or only mildly reduced mitochondrial oxidative enzyme reactivities in-keeping with a mild mitochondrial defect.

In previous studies, we showed that SLC25A1 deficiency results in a primary presynaptic defect [6]. Mitochondria play important roles in the presynaptic nerve terminal, which is a site of high energy requirement [15]. In particular, mitochondria are involved in the regulation of presynaptic calcium [16]. Lambert Eaton Myasthenic Syndrome (LEMS) is caused by antibodies against the presynaptic voltage gated calcium channel. Another CMS phenotype overlapping with LEMS, which results in impaired presynaptic calcium signalling, has been described recently [17]. This includes increment on RNS after a period of maximum voluntary contraction, and response to 3,4-diaminopyridine (3,4-DAP), a presynaptic potassium channel blocker. However, none of the patients in this cohort exhibited features in-keeping with LEMS on neurophysiological tests. 3,4-DAP resulted in good clinical benefit in one case, but has not been trialled in other cases.

In summary, the p.(Arg247Gln) *SLC25A1* variant should be considered in patients presenting with a presynaptic CMS, particularly with accompanying intellectual disability.
